# Apolipoprotein A1 -75 G/A and +83 C/T polymorphisms and renal cancer risk

**DOI:** 10.1186/s12944-015-0132-0

**Published:** 2015-11-04

**Authors:** ZhiHong Liu, YingMing Xiao, LiangYou Tang, Liang Jiang, YuJie Wang, RuoChen Zhang, Qiang Wei, YiPing Lu

**Affiliations:** Department of Urology, West China Hospital, Sichuan University, No 37, Guo Xue Xiang, Chengdu, 610041 China; Department of Urology, The Second people’s Hospital of Sichuan, Chengdu, 610041 China

**Keywords:** Apolipoprotein A1, Renal cancer, Gene polymorphism

## Abstract

**Background:**

Apolipoprotein A1 (ApoA1) is the major apoprotein constituent of high-density lipoprotein that can play important roles in tumor invasion and metastasis. The objective of the present study was to evaluate the association of two genetic variants (−75 G/A and +83 C/T) of *APOA1* with predisposition to renal cancer.

**Methods:**

A total of 432 subjects, including 216 pathologically-proven renal cancer cases and 216 age- and gender-matched healthy controls, were recruited into this hospital-based case–control study. Genotyping of the *APOA1* was performed by polymerase chain reaction-restriction fragment length polymorphism (PCR-RFLP) combined with gel electrophoresis, and then confirmed by direct sequencing.

**Results:**

Patients with renal cancer had a significantly higher frequency of *APOA1* -75 AA genotype [odds ratio (OR) = 2.10, 95 % confidence interval (CI) = 1.18, 3.75; *P* = 0.01] and *APOA1* -75 A allele (OR =1.40, 95 % CI = 1.05, 1.87; *P* = 0.02) than controls. When stratifying by the distant metastasis status, patients with distant metastasis had a significantly higher frequency of *APOA1* -75 AA genotype genotype (OR =2.20, 95 % CI = 1.04, 4.68; *P* = 0.04).

**Conclusion:**

This study is, to our knowledge, the first to examine prospectively an increased risk role of *APOA1* -75 AA genotype and *APOA1* -75 A allele in renal cancer susceptibility.

## Introduction

Renal cancer is the predominant form of malignancy of the kidney and represents 3–4 % of all cancers [1]. In 2015, an estimated 61,560 new cases of kidney and renal pelvis cancers will be diagnosed in the United States [1]. An estimated 14,080 Americans are expected to die from the disease in 2015 [1]. The crude incidence rates of renal cell carcinoma were 7.1 and 3.1 per 100,000 population for men and women respectively in Japan in 1997 [2]. The crude incidence rates of renal cell carcinoma were increased from 4.5 to 5.6 per 100,000 population in China between 1989 and 2008 [3, 4]. Many epidemiological studies have found that environmental factors, such as smoking, diesel exhaust, and various dioxins, may be involved in the development of sporadic renal cancer [5–8]. Although many subjects are exposed to these risk factors during their lifetime, only some of them develop renal cancer, which suggests that genetic susceptibility may play a role in the etiology of renal cancer [9]. The impact of genetic background on renal cancer is still unclear.

Apolipoprotein A1 (ApoA1) is the major apoprotein constituent of high-density lipoprotein (HDL) that can play important roles in tumor invasion and metastasis [10, 11]. Recent findings revealed the crucial roles of ApoA1 in inflammation, tumor growth, angiogenesis, invasion and metastasis [12–14]. *APOA1* gene, located on the 11q23-q24, encodes apoA1 [11, 15]. There are several single-nucleotide polymorphisms (SNPs) in the *APOA1* gene [16]. Two SNPs (−75 G/A and +83 C/T) of *APOA1* play an important role in lipid metabolism [17–19]. It has also been found that *APOA1* -75 G/A and +83 C/T genotypes were associated with susceptibility to breast cancer and lymph node metastases occurrence, respectively [20].

To our best knowledge, no reports concerning the role of *APOA1* -75 G/A and +83 C/T genotypes on renal cancer risk have been reported yet. We hypothesized that *APOA1* -75 G/A and +83 C/T genotypes were associated with renal cancer risk. To test this hypothesis, we performed a prospective hospital-based case–control study to evaluate the association of the *APOA1* -75 G/A and +83 C/T genotypes with predisposition to renal cancer.

## Materials and methods

### Study population

A total of 432 subjects, including 216 pathologically-proven renal cancer cases and 216 age- and gender-matched healthy controls, were recruited into this hospital-based case–control study between February 2012 and December 2014 in the West China Hospital of Sichuan University [21]. All renal cancer cases were histopathologically confirmed. We extracted the following information: tumor grade, tumor classification, lymph node invasion status, distant metastasis status and the pathology of renal cancer. In order to confirm that healthy controls were healthy and free of cancer, volunteers underwent various tests that included physical exams, questionnaires about their health and history, chest X-rays, blood and urine tests for various tumor markers, abdominal ultrasound, gastric endoscopy, and colon enema. The patient or their families/surrogates were interviewed. The Institutional Ethical Committee of the West China Hospital of Sichuan University approved all parts of the study, and informed consent according to the Declaration of Helsinki was obtained from all participants or their families/surrogates.

### DNA extraction and genotyping

**Fig. 1:**
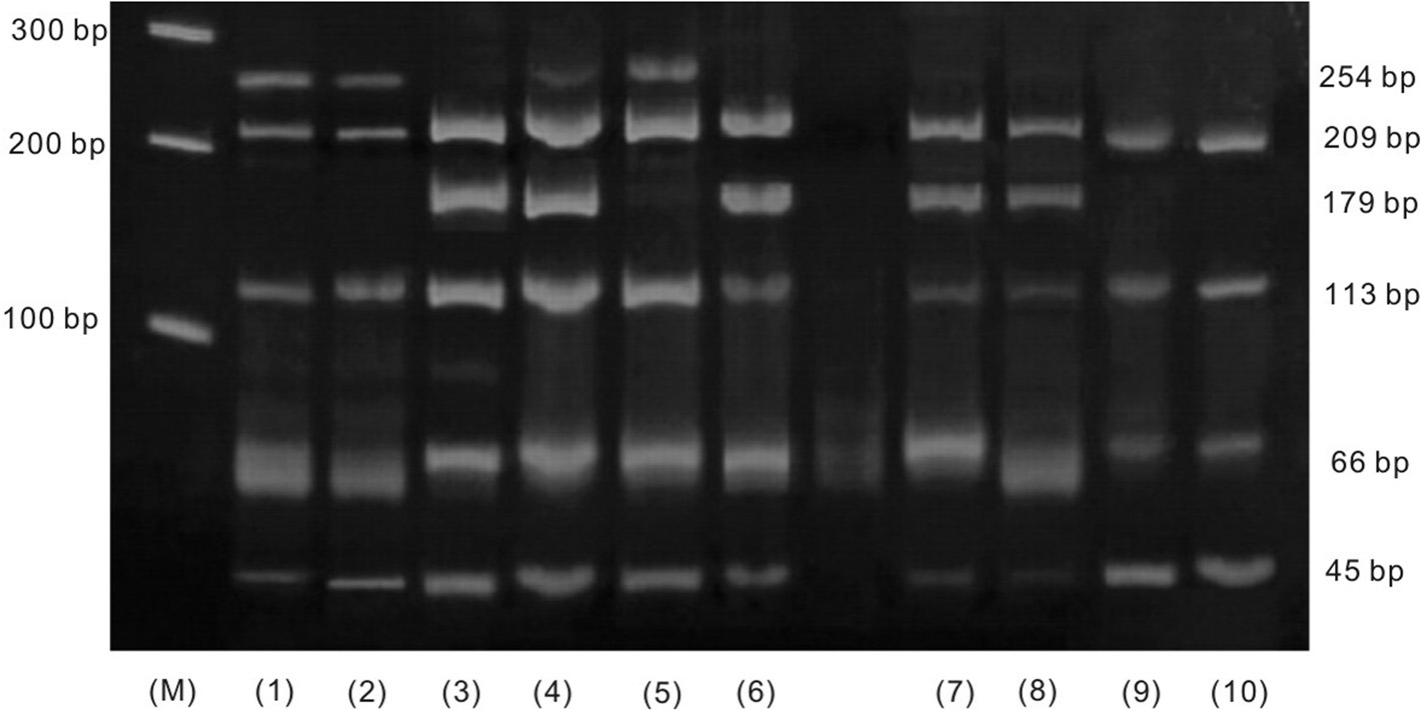
Electrophoresis in a 3 % agarose gel after the *Msp*l digested in renal cancer cases and healthy controls

Genomic DNA was isolated from 20 g/L ethylenediaminetetraacetic acid (EDTA) or sodium citrate anticoagulated 3–5 ml venous blood by the commercially available Qiagen kit (QIAGEN Inc., Valencia, CA, USA) and stored at 4 °C. Genotyping of the *APOA1* was performed by polymerase chain reaction-restriction fragment length polymorphism (PCR-RFLP). Based on the GenBank reference sequence, the PCR primers designed for the *APOA1* -75 G/A and +83 C/T were as follows: 5ʹ-AGG GAC AGA GCT GAT CCT TGA ACT CTT AAG-3ʹ (forward) and 5ʹ-TTA GGG GAC ACC TAG CCC TCA GGA AGA GCA-3ʹ (reverse). The restriction endonuclease enzyme *Msp*I digested the amplified PCR products overnight. Electrophoresis in a 3 % agarose gel followed by ethidium bromide staining and ultraviolet illumination allowed detection of the alleles. The presence of the *Msp*I restriction stie at −75 bp (G allele) and at +83 bp (C allele) in the 433 bp product resulted in four fragments of 45, 66, 113 and 209 bp (Fig. 1). The absence of the restriction site at −75 bp (A allele) resulted in three fragments of 45, 179 and 209 bp (Fig. 1). The absence of the restriction site at +83 bp (T allele) created a larger fragment of 254 bp instead of two fragments of 45 and 209 bp (Fig. 1). For quality control, two independent observers randomly chose 44 samples (22 cases and 22 controls) by computer-generated number scheme. They performed double sampling PCR-RFLP and found no differences, and then confirmed by direct sequencing from Qiagen cleaned up DNA.

### Statistical analysis

Data are presented as percentages for categorical variables or as means ± standard deviation (SD). Differences between categorical variables were evaluated using Pearson *x*^2^ test, while those between continuous variables were assessed by Student’s *t* test. The existence of differences in genotypic frequencies between groups was assessed by means of Pearson *x*^2^ test and calculating the odds ratio (OR) with the 95 % confidence intervals (CI). A *P*-value was considered significant at a level of < 0.05. The Statistical Analysis System software (Version 9.1; SAS Institute Inc., Cary, NC, USA) was used for all statistical tests.

## Results

### Characteristics of participants

Healthy volunteers and cancer patients were not significantly different in terms of age distribution and gender (Table 1). For renal cancer cases, the tumor grade, tumor classification, lymph node invasion status, distant metastasis status and pathology were presented in a separate paper [21].

**Table 1:** General characteristics of renal cancer cases and healthy controls

Variables	Cases	Controls	*P*
Number of subjects	216	216	
Gender (Men/Women)	165/135	163/137	0.87
Age (years)	43.6 ± 9.1	44.1 ± 9.3	0.51
Grade			
1	55		
2	129		
3 + 4	32		
Tumor classification			
T1	103		
T2	49		
T3	57		
T4	7		
Lymph node invasion status			
Negative	199		
Positive	17		
Distant metastasis status			
Negative	188		
Positive	28		
Pathology			
Clear cell carcinoma	197		
Granular cell carcinoma	15		
Chromophobe cell carcinoma	4		

Abbreviations: *SD*, standard deviation

### APOA1 -75 G/A polymorphisms and renal cancer

Patients with renal cancer had a significantly higher frequency of *APOA1* -75 AA genotype [odds ratio (OR) = 2.10, 95 % confidence interval (CI) = 1.18, 3.75; *P* = 0.01] and *APOA1* -75 A allele (OR =1.40, 95 % CI = 1.05, 1.87; *P* = 0.02) than controls (Table 2). When stratifying by the distant metastasis status, patients with distant metastasis had a significantly higher frequency of *APOA1* -75 AA genotype genotype (OR =2.20, 95 % CI = 1.04, 4.68; *P* = 0.04) (Table 3). When stratifying by the tumor grade, tumor classification, lymph node invasion status and pathology, no significant differences were found (Table 3).

**Table 2:** Genotype and allele frequencies of *APOA1* gene polymorphisms (−75 G/A and +83 C/T) among renal cancer cases and healthy controls

Genotypes	Cases (%)	Controls (%)	OR (95 %CI)	*P*
− 75 GG	108 (50.0)	119 (55.1)	1.00 (Reference)	
− 75 GA	66 (30.6)	75 (34.7)	0.97 (0.64,1.48)	0.89
− 75 AA	42 (19.4)	22 (10.2)	2.10 (1.18,3.75)	0.01
−75 G allele frequency	282 (65.3)	313 (72.5)	1.00 (Reference)	
−75 A allele frequency	150 (34.7)	119 (27.5)	1.40 (1.05,1.87)	0.02
+ 83 CC	145 (67.1)	136 (63.0)	1.00 (Reference)	
+ 83 CT	45 (20.8)	49 (22.7)	0.86 (0.54,1.38)	0.53
+ 83 TT	26 (12.1)	31 (14.3)	0.79 (0.44,1.39)	0.41
+83 C allele frequency	335 (77.5)	321 (74.3)	1.00 (Reference)	
+83 T allele frequency	97 (22.5)	111 (25.7)	0.84 (0.61,1.15)	0.27

**Table 3:** Stratification analysis of *APOA1* -75 G/A polymorphisms in renal cancer cases

	Cases	GG			GA			AA		
		*n* (%)	OR (95 %CI)	*P*	*n* (%)	OR (95 %CI)	*P*	*n* (%)	OR (95 %CI)	*P*
Grade	216	108 (50.0)	1 (Reference)		66 (30.6)	1 (Reference)		42 (19.4)	1 (Reference)	
1	55	28 (50.9)	1.02 (0.61,1.70)	0.95	15 (27.3)	0.89 (0.47,1.68)	0.73	12 (21.8)	1.12 (0.55,2.27)	0.75
2	129	64 (49.6)	0.99 (0.68,1.45)	0.97	42 (32.6)	1.07 (0.68,1.66)	0.78	23 (17.8)	0.92 (0.53,1.59)	0.76
3 + 4	32	16 (50.0)	1.00 (0.53,1.90)	1.00	9 (28.1)	0.92 (0.42,2.03)	0.84	7 (21.9)	1.13 (0.47,2.72)	0.79
Tumor classification	216	108 (50.0)	1 (Reference)		66 (30.6)	1 (Reference)		42 (19.4)	1 (Reference)	
T1	103	55 (53.4)	1.07 (0.72,1.59)	0.75	29 (28.2)	0.92 (0.56,1.51)	0.75	19 (18.4)	0.95 (0.53,1.71)	0.86
T2	49	22 (44.9)	0.90 (0.52,1.56)	0.70	16 (32.7)	1.07 (0.57,2.00)	0.84	11 (22.4)	1.16 (0.56,2.40)	0.70
T3	57	28 (49.1)	0.98 (0.59,1.63)	0.95	19 (33.3)	1.09 (0.61,1.96)	0.77	10 (17.6)	0.90 (0.43,1.91)	0.79
T4	7	3 (42.8)	0.86 (0.22,3.38)	0.83	2 (28.6)	0.94 (0.19,4.61)	0.93	2 (28.6)	1.47 (0.29,7.32)	0.64
Lymph node invasion status	216	108 (50.0)	1 (Reference)		66 (30.6)	1 (Reference)		42 (19.4)	1 (Reference)	
Negative	199	99 (49.7)	0.99 (0.71,1.39)	0.98	61 (30.7)	1.00 (0.67,1.49)	0.99	39 (19.6)	1.01 (0.63,1.62)	0.97
Positive	17	9 (52.9)	1.06 (0.46,2.45)	0.89	5 (29.4)	0.96 (0.34,2.71)	0.94	3 (17.7)	0.91 (0.26,3.24)	0.88
Distant metastasis status	216	108 (50.0)	1 (Reference)		66 (30.6)	1 (Reference)		42 (19.4)	1 (Reference)	
Negative	188	99 (52.7)	1.05 (0.75,1.47)	0.76	59 (31.4)	1.03 (0.69,1.54)	0.90	30 (15.9)	0.82 (0.49,1.36)	0.45
Positive	28	9 (32.1)	0.64 (0.29,1.41)	0.27	7 (25.0)	0.82 (0.34,1.96)	0.65	12 (42.9)	2.20 (1.04,4.68)	0.04
Pathology	216	108 (50.0)	1 (Reference)		66 (30.6)	1 (Reference)		42 (19.4)	1 (Reference)	
Clear cell carcinoma	197	99 (50.2)	1.01 (0.72,1.40)	0.98	60 (30.5)	1.00 (0.67,1.49)	0.99	38 (19.3)	0.99 (0.61,1.60)	0.97
Granular cell carcinoma	15	7 (46.7)	0.93 (0.37,2.36)	0.88	5 (33.3)	1.09 (0.38,3.11)	0.87	3 (20.0)	1.03 (0.29,3.71)	0.97
Chromophobe cell carcinoma	4	2 (50.0)	1.00 (0.18,5.55)	1.00	1 (25.0)	0.82 (0.09,7.45)	0.86	1 (25.0)	1.29 (0.14,11.79)	0.82

### APOA1 + 83 C/T polymorphisms and renal cancer

We did not find any association between *APOA1* + 83 C/T polymorphisms and renal cancer risk (Table 2).

## Discussion

Many studies have suggested that genetic susceptibility may play a role in the etiology of renal cancer. The DKK3 polymorphisms were associated with renal cancer and that the DKK2 rs17037102 polymorphism might be a predictor for survival in patients with renal cancer after radical nephrectomy [22]. We recently found that *IL-6* -174 CC genotype was associated with an increased risk for renal cancer [21]. The functional -31G/C polymorphism in the promoter of survivin might influence the susceptibility and progression of renal cancer in the Chinese population [23]. The polymorphisms of the *CYP1B1* gene at codons 119 and 432 might be risk factors for renal cancer, especially in the male population [7]. The *CYP1A1* polymorphisms might play an important role in the etiology of renal cancer [24]. The polymorphisms of catechol-O-methyltransferase in men were associated with renal cancer [25]. The R allele of paraoxonase-1 gene Q192R polymorphism might protect against renal cancer [26]. Two SNPs in *AGTR1* might be a candidate pathway in renal cancer etiology [27]. Polymorphisms in genes of the renin-angiotensin-aldosterone system (*AGTR1* and *AGT*) influenced renal cell cancer susceptibility [27]. A common variant, rs35252396, at 8q24.21 was associated with renal cell cancer [28].

The *APOA1* gene polymorphisms were also associated with many other diseases. It has been found that the *APOA1* -75 G/A polymorphism was associated with gallstone disease [29]. The *APOA1* -75 G/A and +83 C/T genotypes were also associated with susceptibility to breast cancer and lymph node metastases occurrence, respectively [20]. A pilot study found that *APOA1* polymorphisms (−75 G/A and +83 C/T) might be susceptibility to myocardial infarction in a north Indian population [19]. The individuals with the APOA1 -75 A allele were likely to have a lower risk of coronary artery disease as a result of its effect on higher serum concentrations of ApoA1 and HDL-C [30]. The *APOA1* -75G/A promoter polymorphism was associated with cognitive performance in multiple sclerosis [31]. It has been found that the *APOA1* polymorphisms (−75 G/A and +83 C/T) could be as risk factors for hypertension and obesity in a Brazilian elderly cohort [32]. The *APOA1* -75 A allele was associated with an increased risk for Alzheimer’s disease [33]. The *APOA1* -75 AA genotype was associated with a higher acute lung injury risk after cardiopulmonary bypass surgery [34].

The exact biological mechanism of the association between the *APOA1* -75G/A polymorphisms and the risk of renal cancer is still unclear. ApoA1 can play important roles in tumor growth, angiogenesis, invasion and metastasis [12–14]. Expression of ApoA1 is associated with colonic adenocarcinoma progression, and thus ApoA1 is a potential marker of the aggression [35]. It has also been found that *APOA1* -75 G/A and +83 C/T genotypes were associated with susceptibility to breast cancer and lymph node metastases occurrence, respectively [20]. A recent fascinating study reveals an overall protective ability of HDL, specifically *APOA1*, to induce tumor suppression through both innate and adaptive immune processes in multiple animal tumor models [36]. Tabet et al*.* demonstrated that HDL’s anti-inflammatory properties were conferred, in part, through HDL-micro-RNA (miR)-223 delivery and translational repression of ICAM-1 in endothelial cells [37]. However, the miR221/222 cluster increases the aggressiveness of tumors in epithelial cancers, through repression of tumor suppressors and through induction of cell motility [38]. *APOA1* allelic variety may have an impact on angiogenesis [39]. *APOA1* binding protein (AIBP) positively regulates cholesterol efflux from endothelial cells and that effective cholesterol efflux is critical for proper angiogenesis [39]. AIBP is highly expressed in human renal cancer and in 83 % of cancers in general [39]. But there is no study about the interaction of AIBP and the *APOA1* alleles. Further research on the possible interaction of AIBP and the APOA1 alleles is necessary.

Some shortcomings of this study should be mentioned. First of all, potential selection bias might have been present, because this is a hospital based case control study and the subjects may not be representative of the general population. Second, this study is limited by its size and lack of replication. Finally, further research on the biological mechanism of the association between the *APOA1* -75G/A polymorphisms and the risk of renal cancer is necessary.

In conclusion, to our best knowledge, up to now this study is the first to examine prospectively an increased risk role of *APOA1* -75 AA genotype and *APOA1* -75 A allele in renal cancer susceptibility.
